# Predictive role of endothelial cell activation in cytokine release syndrome after chimeric antigen receptor T cell therapy for acute lymphoblastic leukaemia

**DOI:** 10.1111/jcmm.17029

**Published:** 2021-11-03

**Authors:** Fei Hong, Ming Shi, Jiang Cao, Ying Wang, Yanqing Gong, Hui Gao, Zhenyu Li, Junnian Zheng, Lingyu Zeng, Aili He, Kailin Xu

**Affiliations:** ^1^ Department of Hematology The Affiliated Hospital of Xuzhou Medical University Jiangsu China; ^2^ Department of Hematology The Second Affiliated Hospital of Xi’an Jiaotong University ShaanXi China; ^3^ Cancer Institute Xuzhou Medical University Jiangsu China; ^4^ Jiangsu Bone Marrow Stem Cell Institute Jiangsu China

**Keywords:** acute lymphoblastic leukaemia, biomarker, chimeric antigen receptor T cell, cytokine release syndrome, endothelial cell

## Abstract

CD19‐target chimeric antigen receptor (CAR)‐T cell therapy is highly effective for relapsed/refractory (R/R) acute lymphoblastic leukaemia (ALL), but is often complicated by cytokine release syndrome (CRS), which is potentially life‐threatening. Endothelial cells are the core regulator of CRS in many infectious diseases and may also play a key role after CAR‐T cell therapy. We provided a detailed clinical, laboratory description and endothelial cell activation biomarkers in patients with CRS. Endothelial cell activation was associated with occurrence, development and severity of CRS, manifested by decreased serum angiopoietin (Ang)‐1 levels and increased levels of von Willebrand Factor (VWF), Ang‐2, Ang‐2:Ang‐1, sE‐selectin, soluble intercellular adhesion molecule (sICAM‐1) and soluble vascular cell adhesion molecule (sVCAM)‐1. Besides, the endothelial activation was correlated with the hepatic, kidney and hematopoietic dysfunction in CRS patients. After infusion of CAR‐T cells, we detected changes of endothelial activation‐related biomarkers within 36 hours in patients who subsequently developed CRS, especially severe CRS. Using top tree models, we could predict which patients would develop CRS, especially severe CRS, or identify the severity of CRS by certain biomarkers of endothelial activation. These data provide a new idea and will help identify new targets for early intervention and prevention of CRS.

## 1 INTRODUCTION

Chimeric antigen receptor T (CAR‐T) cell therapy is a novel immunotherapy for the treatment of highly refractory haematologic malignancies. CAR‐T cells specific for CD19 have shown good efficacy and encouraging results in the treatment of relapsed and/or refractory (R/R) CD19 B cell acute lymphoblastic leukaemia (B‐ALL),^1^ but they also come with serious and potentially life‐threatening complications.^2^ After infusion of CAR‐T cells, they are activated by target tumour cells, leading to proliferation of CAR‐T cells, cleavage of target cells and secretion of cytokines including inflammatory cytokines, which results in complications such as cytokine release syndrome (CRS).^3^

As the most common potentially severe toxicity of CAR‐T cell therapy, CRS is characterized by acute systemic inflammation, elevated levels of circulating cytokines, as well as secondary organ dysfunction, such as liver and renal impairment and hematopoietic toxicity. Especially, severe CRS could be life‐threatening, becoming a big obstacle to CAR‐T cell therapy.^4^ Therefore, how to break through CRS and improve the success rate of CAR‐T cell therapy is an urgent problem to be solved.

CRS involves a complex, interconnected network of multiple cytokines and immune cells, in which interferon(IFN)‐γ, interleukin(IL)‐1, IL‐6, IL‐18 and tumour necrosis factor (TNF) are key cytokines, monocytes and macrophages are the main driver cells reported in recent studies.^5^ Currently, interest has focused on the role of the endothelial cells in CRS. After CAR‐T cell infusion, patients with severe CRS often have vascular instability, capillary leakage and consumptive coagulation disorders, suggesting that endothelial cell may be the core regulator of CRS.^6^

Here, we report the clinical and laboratory findings from patients with CD19 refractory/relapsed ALL after receiving CAR‐T cell therapy. We measured 6 biomarkers of endothelial cell activation and several other laboratory markers. We also identified risk factors associated with the incidence and severity of subsequent CRS from activated endothelial cells. Using the biomarkers of endothelial cell activation, we could identify patients at high risk of CRS early in CAR‐T cell therapy, especially those with severe CRS, and initiate early interventions for them, which would help to improve the success rate of CAR‐T cell therapy.

## 2 METHODS

### 2.1 Patients and study design

We collected clinical and laboratory data of 30 patients with ALL who received CD19 CAR‐T cell immunotherapy in the Affiliated Hospital of Xuzhou Medical University from October 2017 to August 2020. The study was approved by the Ethics Committee of Xuzhou Medical University Affiliated Hospital. All patients signed informed consent. CRS is defined, graded and managed according to the current concept of diagnosis and management of cytokine release syndrome proposed by used the Lee, et al. in 2014.^7^ For patients who developed fever, start of CRS was defined as the day with the first fever ≥38.0℃ relative to infusion of CD19 CAR‐T cells. Stop of CRS was defined as 24 hours without fever or vasoactive medications, indicating recovery from shock. For two patients developed CRS without fever, start and stop of CRS were defined based on the first day with flu‐like symptoms and the first 24‐hour period without symptoms, respectively. CRS was assessed by three experienced clinicians and was reassessed if there is disagreement.

### 2.2 CD19 CAR‐T cell infusion

Peripheral blood mononuclear cells (PBMCs) were obtained from patients by leukapheresis for CD19 CAR‐T cells preparation on day −11, and the first day of CAR‐T cells infusion was set on day 0. The CD19 CAR‐T cells were acquired through the transfection of lentivirus carrying a CAR sequence of humanized anti‐CD19 scFv into peripheral CD3^+^ T cells. The design of the CAR transgene and CAR‐T cell manufacturing have been previously described.^8^ A truncated human epidermal growth factor receptor (EGFRt) was encoded in the lentiviral vector to allow precise enumeration of transduced CAR‐T cells by flow cytometry.^9^ Most patients received lymphodepletion chemotherapy with FC regimen consisting of fludarabine (30 mg/m^2^/day, days −5 to −2) and cyclophosphamide (750 mg/m^2^, day −5), while others received cyclophosphamide‐based lymphodepletion chemotherapy regimens. The date of CAR T cells infusion was set as day 0. On day 0, patients with ALL received a single dose of CD19 CAR‐T cell infusion at 1 × 10^6^ EGFRt^+^ cells/kg.

### 2.3 Collection of clinical and laboratory data

Peripheral blood was collected before lymphodepletion, on day −1 or day 0, and approximately 1, 2, 3, 5, 7, 10, 14, 21 and 28 days after CAR‐T cell infusion for analyses of soluble adhesive molecules, angiopoietin (Ang)‐1, Ang‐2, von Willebrand Factor (VWF), peripheral blood cells, renal and hepatic function. Patients were closely monitored until 30 days after CAR‐T cell infusion. The soluble adhesive molecules (sE‐selectin, sVCAM‐1 and sICAM‐1), ANG‐1, ANG‐2 and VWF concentration were measured by Luminex assay.

### 2.4 Luminex assay

Human cytokine magnetic 7‐plex panel was purchased from R&D Systems (catalog number: LXSAHM‐07). The following analytes are in the panel: VWF, Ang‐2, Ang‐1, sE‐selectin, sVCAM‐1, sICAM‐1 and SCF. Human cytokine magnetic 1‐plex (SDF‐1) was also purchased from R&D Systems (catalog number: LXSAHM‐01). Serum samples cryopreserved at −80°C from day −5 to day 30 were thawed and analysed according to the manufacturers’ protocols. Assay plates were measured using a FlexMAP 3D instrument (Luminex, Austin, TX), and data acquisition and analysis were done using xPONENT software (Luminex). Data quality was examined based on the following criteria. The standard curve for each analyte has a R^2^ value >0.99 with or without minor fitting using xPONENT software. Samples with results that were out of range low were re‐tested and substituted with half of the lower end of the standard curve for data analysis. Samples with results that were out of range high or greater than two times the standard curve maximum value were re‐tested at higher dilutions.

### 2.5 Statistical analyses

Clinical, laboratory and other biomarkers associated with CRS were comprehensively reviewed and summarized according to the occurrence of CRS in patients. For indicators monitored continuously, their values were summarized at day 3 and 1 month after infusion, respectively, in order to obtain early and overall peaks during CRS in patients. Measurement data were expressed as mean ± standard error (SEM). Log10 values were used to transform data as appropriate. For continuous variables between the two groups, Student's t test was used for homogeneity of variance, while Wilcoxon test or Mann‐Whitney test was used for heterogeneity of variance. Values below the detection lower limit are recorded as half of the lower limit. All statistical analyses were performed using IBM SPSS for Windows 25.0 software, and *p* < 0.05 was considered to be statistically significant.

## 3 RESULTS

### 3.1 Clinical Description of Patients

Thirty patients with B‐ALL, who received infusion of CD19‐targeted CAR‐T cells, were included in our study. Patient characteristics were summarized in Table 1. The median age was 21 years (range 6 to 69). Three (10%) patients had undergone hematopoietic stem cell allogeneic transplantation (allo‐HSCT) previously. 28 (93%) patients received lymphodepletion chemotherapy with FC regimen, and 2 (7%) patients received cyclophosphamide‐based lymphodepletion chemotherapy regimens, including CHOP and COP (Table 1).

**TABLE 1:** Patient characteristics

Characteristics	N = 30
Age (years)	
Median (range)	21 (6–69)
Sex	
Male	12 (40%)
Female	18 (60%)
BM Blasts of ALL	
<5%	9 (30%)
≥5%	21 (70%)
Transplant history, n (%)	
Allo‐HSCT	3 (10%)
No	27 (90%)
Lymphodepletion regimen, n (%)	
Cy/Flu	28 (93%)
Non‐Cy/Flu	2 (7%)
Cytokine release syndrome, n (%)	
Grade 1	11 (37%)
Grade 2	10 (33%)
Grade 3	3 (10%)
Grade 4	5 (17%)
Grade 5	1 (3%)

### 3.2 Laboratory Description of Cytokine Release Syndrome (CRS)

CRS was graded according to consensus criteria proposed by Lee et al.^6^ In patients with CRS after CAR‐T cell infusion, a majority of patients (24/30; 80%) had grades 1–3 CRS (grade 1, 37%; grade 2, 33%; and grade 3, 10%), six patients (20%) developed grade ≥4 CRS (grade 4, 17% and grade 5, 3%), and the patient with grade 5 died on day 11 after CAR‐T cell infusion (Table 1).

We conducted a series of assessments on laboratory markers of inflammation and organ dysfunction in patients treated with CD19‐targeted CAR‐T cells (Table 2). Baseline ferritins (N = 29) were elevated in most patients due to systemic inflammation and/or iron overload. One patient did not have ferritin tested at baseline. Only 2 patients out of the 29 measured had baseline ferritins <150 ng/mL (upper limit of normality). Peak ferritins (defined as the highest value in the first month after CD19‐targeted CAR‐T cells infusion) were very high in all patients (median 2717 ng/mL, range 157.5 – 998997 ng/mL) regardless of grade (*p* < 0.0001), but there was no significant difference between mild (grades 1–3) and severe CRS(grades 4–5) (*p* = 0.0559).

**TABLE 2:** Clinical biomarkers are associated with CRS. Unless otherwise noted, the median (range) for the peak observed value within one month is displayed

Biomarker	Baseline	Peak		
		Total (N = 30)	Grade 1–3 (N = 24)	Grade 4–5 (N = 6)
Ferritin (ng/mL)	921.4 (82.2–5489)	2717 (157.5–99899)****	2030 (157.5–99899)**	15375 (2622–49927)****
CRP (mg/L)	9.8 (0.3–71.0)	118 (1.5–227.7)****	118 (1.5–200)****	139.7 (33.6–227.7)****
IL−6 (pg/mL)	7.35 (1.4–60.0)	359.7 (5.1–5001)****	139.7 (5.1–5001)****	3026 (398.9–5001)****
ALT (U/L)	16.5 (4–90)	34 (8–801)**	32.5 (8–292)*	110.5 (25–801)***
AST (U/L)	19 (5–68)	40.5 (5–3725)***	34.5 (5–298)**	522 (9–3725)*
BUN (mmol/L)	3.8 (1.7–7.9)	5.35 (2.1–16.1)****	5 (2.1–10.3)*	8.2 (5.6–16.1)****
Cr (ummol/L)	43 (14–93)	62.5 (24–314)****	54 (24–314)	83 (45–166)**
SCF nadir (pg/mL)	60.5 (20.3–234.7)	51.4 (16.9–170.2)***	51.4 (16.9–148.4)	48.6 (36.0–170.2)
SDF−1 nadir (pg/mL)	1260 (63.5–2186)	249.6 (63.5–1338)****	276.3 (63.5–2186)****	63.5 (63.5–547.8)***
WBC nadir (10^9^/L)	1.0 (0.1–57.1)	0.7 (0.1–44.5) ****	0.75 (0.1–44.5)	0.15 (0.1–1.9)**
RBC nadir (10^12^/L)	2.63 (1.03–4.66)	1.87 (1.05–4.17)****	2.02 (1.05–4.17)	1.65 (1.33–2.14)**
Hb nadir(g/L)	78.5 (32–127)	52 (33–109)****	59 (33–109)*	51 (40–67)**
PLT nadir(10^9^/L)	70.5 (4–267)	26 (1–170)****	43 (1–170)	6.5 (1–20)***

Abbreviations: ALT = alanine aminotransferase; AST = aspartate aminotransferase; BUN = Blood urea nitrogen; Cr = Creatinine; CRP = C Reactive Protein; SCF = stem cell factor; SDF‐1 = stromal cell–derived factor‐1. The Mann‐Whitney test was used for statistical analysis. Compare with baseline values, *p* < 0.05 means statistically significant

* *p* < 0.05

** *p* < 0.01

*** *p* < 0.001

**** *p* < 0.0001.

Baseline CRP was elevated in most patients (median 9.8 mg/L, range 0.3–71.0 mg/L). One patient was not tested for CRP at baseline. 17 out of 29 patients (59%) had a baseline CRP >5 mg/L (upper limit of normality). Peak CRP in the first month was very high in 97% (29/30) of patients (median 118 mg/L, range 1.5 – 227.7 mg/L) regardless of grade (*p* < 0.0001), with no significant difference in patients between severe and mild CRS (*p* = 0.5258).

Baseline IL‐6 was elevated in half of the patients (median 7.35 pg/mL, range 1.4 – 60.0 pg/mL). 15 out of 30 patients (50%) had a baseline IL‐6 > 7 pg/mL (upper limit of normality). Peak IL‐6 was were very high in 93% (28/30) of patients (median 359.7 pg/mL, range 5.1–5001 pg/mL) regardless of grade (*p* < 0.0001), with a statistically significant increase in severe CRS comparing to mild CRS (*p* < 0.01).

Liver function abnormalities, particularly aspartate aminotransferase increased, were the most common signs of end organ injury among patients with CRS, and kidney abnormalities function were less frequent.^10^ In our study, baseline ALT was elevated (above the upper limit of normal, 40 U/L) in 11% (3/28) of patients (median 16.5 U/L, range 4.0 – 90.0 U/L). Peak ALT was high in 43% (15/30) of patients (median 34 U/L, range 8–801 U/L) regardless of CRS grade (*p* < 0.01), with a statistically significant increase in severe versus mild CRS (*p* < 0.01). Like ALT, baseline AST was also elevated (above the upper limit of normal, 35 U/L) in 11% (3/28) of patients, and peak ALT was very high in 60% (19/30) of patients, regardless of grade (*p* < 0.001). Peak BUN was high in 13% (6/30) of patients (median 34 U/L, range 8– 801 U/L) regardless of grade (*p* < 0.01), with a statistically significant increase in severe versus mild CRS (*p* < 0.01). Peak creatinine (Cr) markedly increased in 27% (10/30) of patients with CRS (*p* < 0.0001) (Table 3). While peak values of these markers were associated with CRS, but only IL‐6, ALT and BUN were correlated with the severity of CRS. The median of AST, ALT, BUN and Cr peaked between days 9 and 11, which were later than CRS (Table 4).

**TABLE 3:** Organ abnormalities among patients with CRS

	Total patients with CRS		
	Total (N = 30)	Grade 1–3 (N = 24)	Grade 4–5 (N = 6)
Liver			
ALT high	15/30 (50%)	10/24 (42%)	5/6 (83%)
AST high	19 /30(63%)	14/24 (58%)	5/6 (83%)
Kidney			
BUN high	6/30 (20%)	3/24 (13%)	3/6 (50%)
Cr high	10/30 (33%)	6/24 (25%)	4/6 (67%)
Blood			
Leukopenia	28/30 (93%)	22/24 (92%)	6/6 (100%)
Erythropenia	28/30 (93%)	22/24 (92%)	6/6 (100%)
Hemoglobinopenia	30/30 (100%)	24/24 (100%)	6/6 (100%)
Thrombocytopenia	26/30 (87%)	20/24 (83%)	6/6 (100%)

Abbreviations: ALT = alanine aminotransferase; AST = aspartate aminotransferase; BUN = Blood urea nitrogen; Cr = Creatinine.

**TABLE 4:** Days to peak/nadir of the relevant parameters after CAR‐T cells infusion

Parameters	Median (Range)
CRS peak	8 (1 – 15)
Endothelial cell activation	
VWF peak	5 (1 – 12)
Ang−1 peak	5 (1 – 19)
Ang−2 peak	6 (1 – 21)
sE‐selectin peak	4.5 (1 – 12)
sVCAM−1 peak	6 (3 – 14)
sICAM−1 peak	4 (2 – 9)
Liver	
ALT peak	9 (1 – 28)
AST peak	9 (1 – 28)
Kidney	
BUN peak	11 (1 – 28)
Cr peak	10 (3 – 29)
Haematopoiesis	
SCF nadir	7 (2 – 19)
SDF−1 nadir	7 (1 – 19)
WBC nadir	6.5 (1 – 20)
RBC nadir	10.5 (1 – 27)
Hb nadir	10 (1 – 27)
PLT nadir	11.5 (2 – 28)

Abbreviations: CRS = Cytokine release syndrome; VWF = von Willebrand factor; Ang‐1 = Angiopoietin‐1; Ang‐2 = Angiopoietin‐2; sICAM‐1 = soluble intercellular adhesion molecule‐1; sVCAM‐1 = soluble vascular cell adhesion molecule‐1; ALT = alanine aminotransferase; AST = aspartate aminotransferase; BUN = Blood urea nitrogen; Cr = Creatinine; SCF = stem cell factor; SDF‐1 = stromal cell–derived factor‐1; WBC = white blood cell; RBC = red blood cell; Hb = haemoglobin; PLT = platelet.

We also evaluated hematopoietic toxicity in patients with CRS after CAR‐T cell infusion, and only patients who received Cy/Flu lymphodepletion (n = 28) were included in this evaluation to ensure the differences in hematopoietic toxicity were not due to lymphodepletion regimen before CAR‐T cell therapy. We detected the levels of SCF (stem cell factor) and SDF‐1 (stromal cell–derived factor‐1) which play the key role on haematopoiesis and found the minimum SCF or SDF‐1 were significantly lower than baseline values. The median of minimum white blood cell (WBC), red blood cell (RBC), haemoglobin concentration (Hb) and platelet (PLT) was low in patients with CRS, especially those with severe CRS, and patients with severe CRS received lower WBC (*p* = 0.019) and PLT (*p* = 0.009) than those with mild CRS (Table 2). The minimum of WBC and RBC was low in 93% (28/30) of patients regardless of CRS grade, the minimum of PLT was low in 87% (26/30) of patients, and the minimum of Hb was low in all patients. The median of SCF, SDF‐1, WBC, RBC, Hb and PLT counts declined after Cy/Flu chemotherapy and CAR‐T cell infusion, reaching their lowest point between days 6.5 and 11.5 after CAR‐T cell infusion (Table 4).

### 3.3 CRS is associated with endothelial cell activation

To clarify the correlation between the activation of endothelial cell and the occurrence of CRS after CAR‐T therapy, we performed serial assessment of endothelial cell‐related biomarkers on the 30 patients. We compared median baseline values from 30 ALL patients with 7 normal donors. Compared to the normal donors, we found that some of the endothelial cell‐related biomarkers, including VWF, Ang‐1: Ang‐2 ratio, sVCAM‐1 and sICAM‐1 were elevated in most patients, while Ang‐1 was declined (Supplemental Table S1). Moreover, these biomarkers were not associated with baseline disease burden, suggesting that the changes of endothelial cell‐related biomarkers occurred after CAR‐T cell infusion.

**FIGURE 1:**
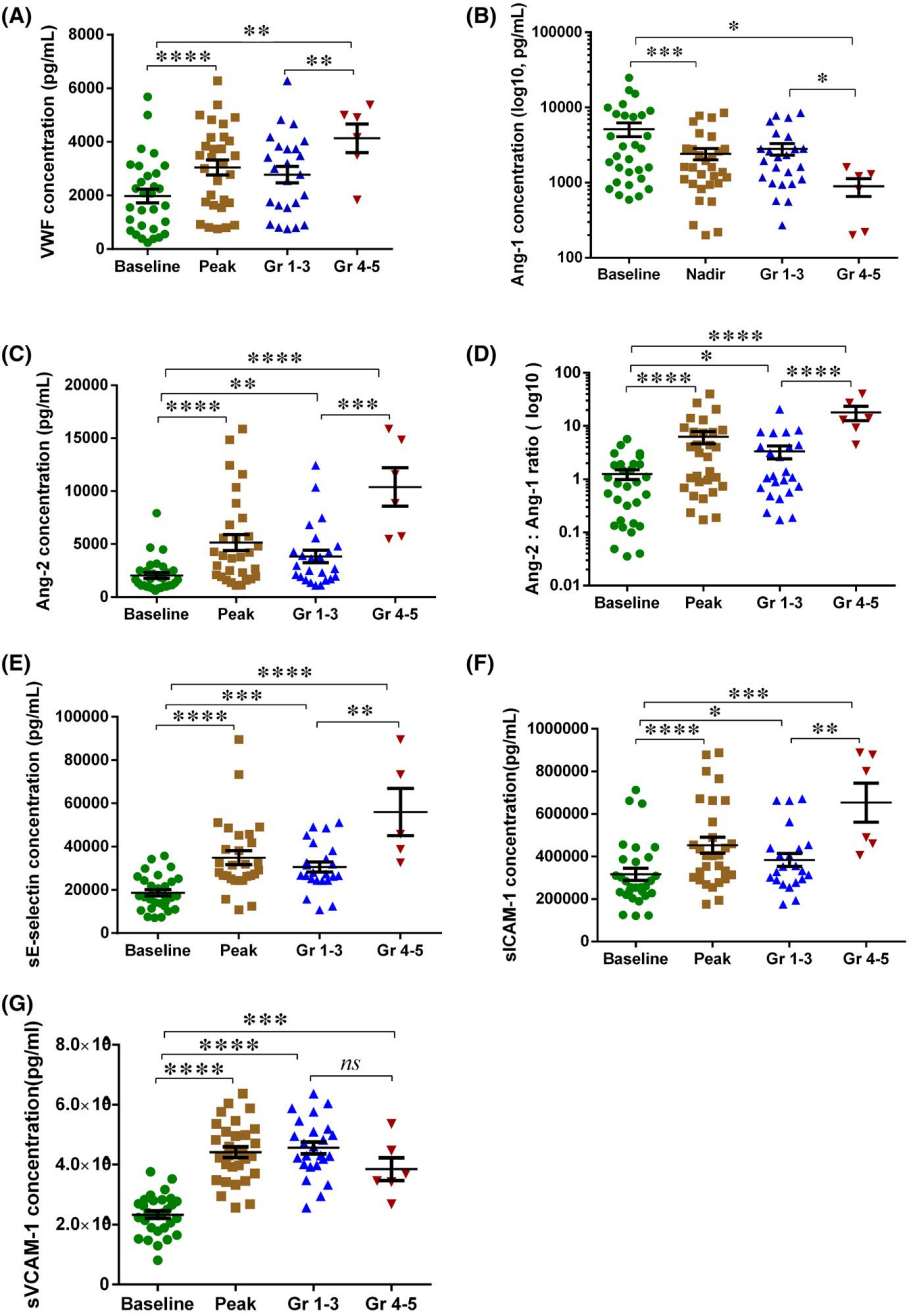
Biomarkers of endothelial activation associated with the occurrence and severity of CRS. Serial assessment of 6 endothelial cell‐related biomarkers was performed in 30 patients treated with CD19‐targeted CAR‐T therapy. The baseline or peak/nadir values of biomarkers over the first month were depicted in Figure 1. VWF (A), Ang‐1 (B), Ang‐2 (Angiopoietin‐2, C), Ang‐2:Ang1 ratio (D), sE‐selectin (E), sICAM‐1(F) and sVCAM‐1(G) values collected approximately 30 days after CAR‐T cell infusion from total patients or a subset of patients with grades 1–3 (n = 24) or grades 4–5 (n = 6) CRS. VWF = von Willebrand factor, Ang‐1 = Angiopoietin‐1, Ang‐2 = Angiopoietin‐2, sICAM‐1 = soluble intercellular adhesion molecule‐1, sVCAM‐1 = soluble vascular cell adhesion molecule‐1. P values were determined using Student's t test for homogeneity of variance or Wilcoxon/Mann‐Whitney test for heterogeneity of variance. ^*^ *p* < 0.05, ^**^ *p* < 0.01, ^****^ < 0.001, ^****^ *p* < 0.0001, ns means *p* < 0.05

**FIGURE 2:**
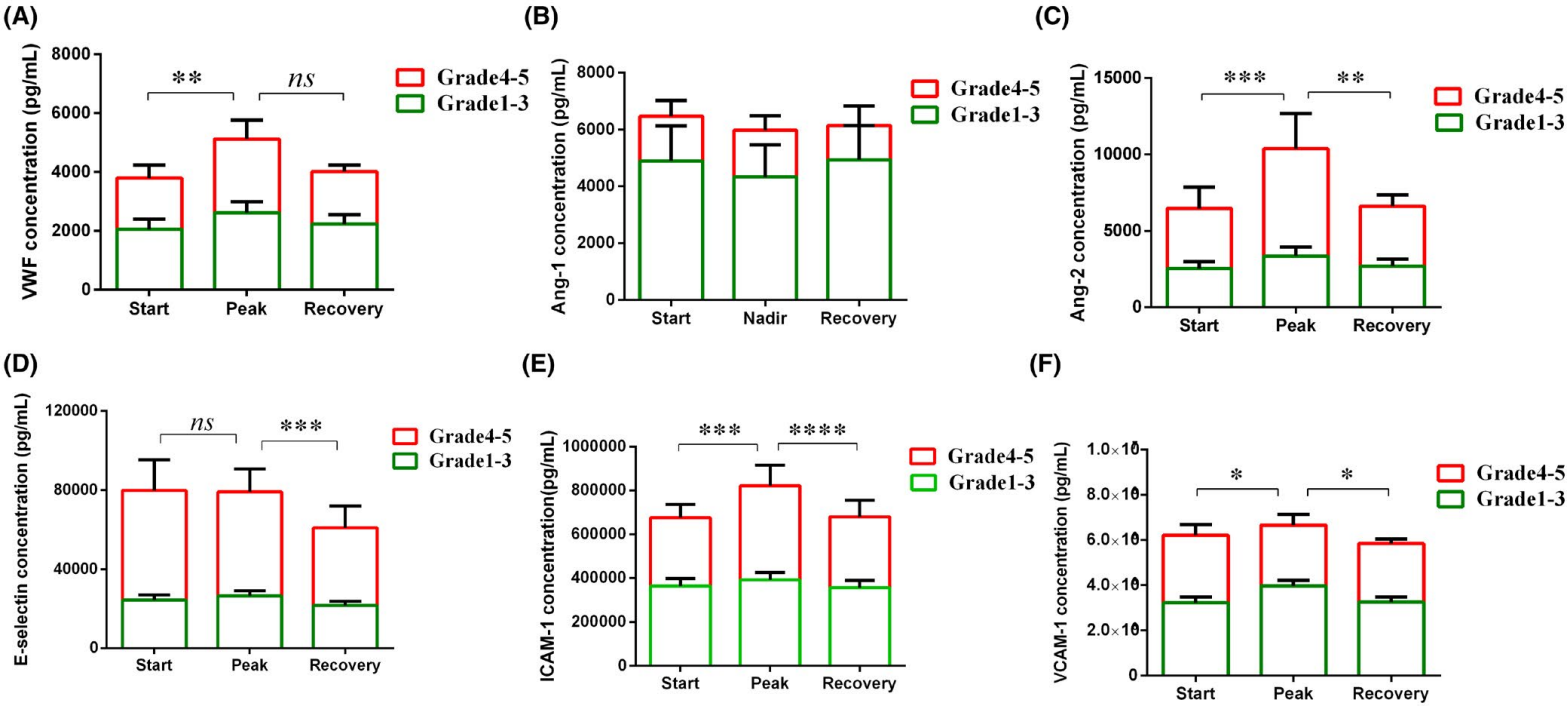
Biomarkers of endothelial activation associated with the development process of CRS. VWF (A), Ang‐1 (B) and Ang‐2 (C) concentrations, sE‐selectin (D), sICAM‐1(E) and sVCAM‐1(F) in serum collected on at the start, peak and stop of CRS after CAR‐T cell infusion from 30 patient. P values were determined using Student's t test or Mann‐Whitney test. *p < 0.05, **p < 0.01, ****<0.001, **** *p* < 0.0001, ns means *p* < 0.05

We compared endothelial cell‐related markers in patients who had severe CRS with those who had mild CRS (Supplemental Table S2). Compared to the patients with mild CRS after CAR‐T cell therapy, we found peak levels of VWF, Ang‐2, Ang‐2:Ang‐1 ratio, sE‐selectin and sICAM‐1 in the first month were higher in patients with severe CRS, while nadir level of Ang‐1 was lower (Figure 1). We also compared the markers at the start, peak and recovery of CRS, and found that peak VWF, Ang‐2, sICAM‐1 and sVCAM‐1 were higher at the peak of CRS than at the start of CRS, while peak VWF, Ang‐1, Ang‐2, Ang‐2:Ang‐1 ratio, sE‐selectin and sICAM‐1 declined when CRS was recovered (Figure 2). These data indicated that biomarkers of endothelial cell activation are not only associated with CRS severity, but also with the development of CRS.

### 3.4 Endothelial cell activation is associated with organ dysfunction

**FIGURE 3:**
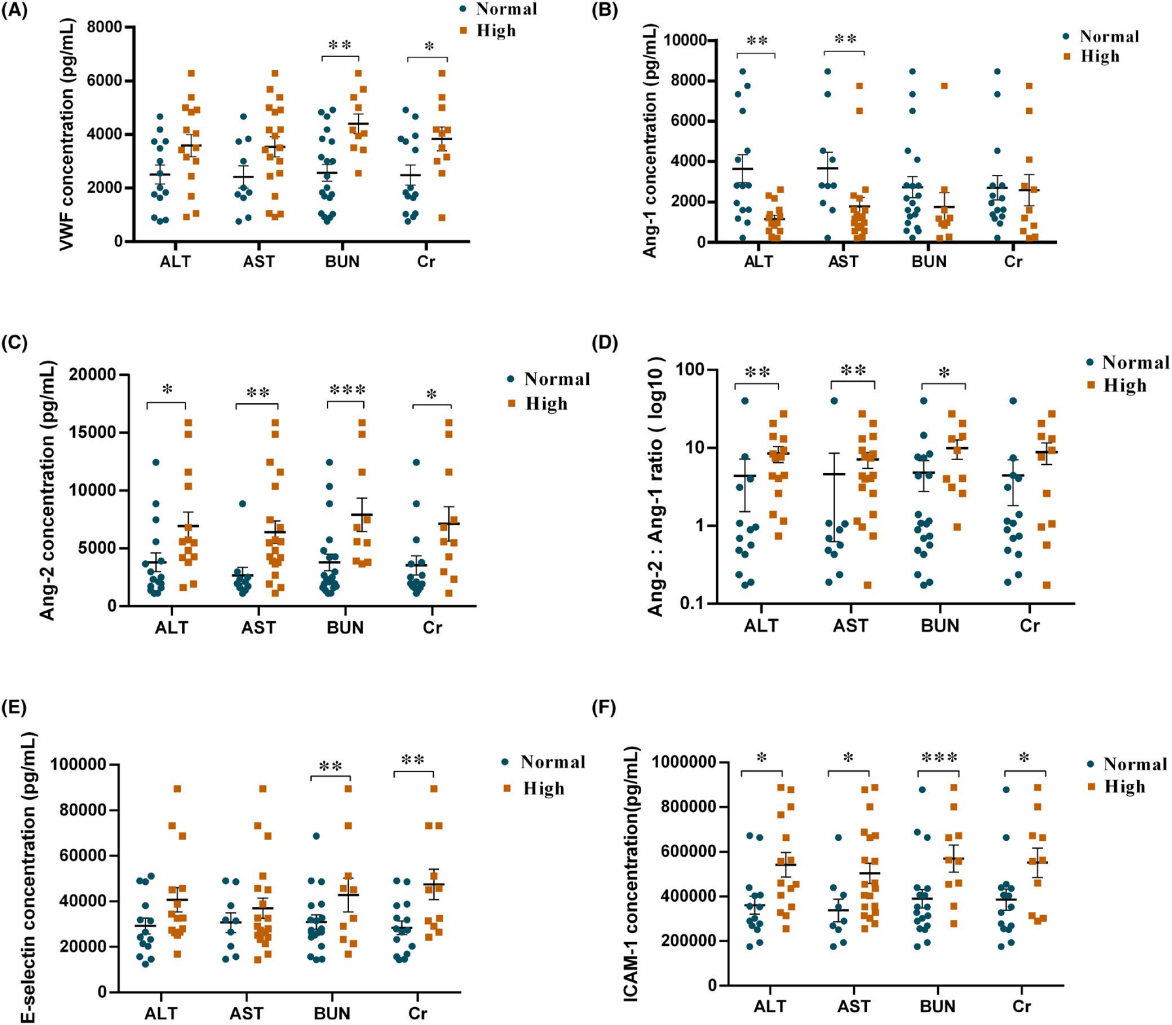
The levels of endothelial activation biomarkers with the normal or high values of liver and kidney dysfunction parameters. Serum peak VWF (A), Ang‐1 (B), Ang‐2 (C), Ang‐2:Ang‐1 ratio (D), sE‐selectin (E), sICAM‐1 (F) in CRS patients with normal or elevated ALT (alanine aminotransferase), AST (aspartate aminotransferase), BUN (blood urea nitrogen) and Cr (creatinine) values. Mean ± SEM values are shown; P values were determined using Mann‐Whitney test, *p < 0.05, **p < 0.01,^****^<0.001,^****^ *p* < 0.0001

Liver and kidney dysfunction was a common sign of organ injury in patients with CRS, but it is not clear who is responsible for this.^10^ Here, we compared biomarkers associated with endothelial cell activation in patients who had liver or kidney function abnormalities with patients who did not (Figure 3). Peak level of VWF in the first month after CAR‐T cell infusion was higher in patients with elevated BUN and Cr, while minimum level of Ang‐1 was lower in patients with elevated ALT and AST. Peak Ang‐2 and sICAM‐1 in the first month after CAR‐T were associated with hepatic and renal dysfunction, manifest by elevated ALT, AST, BUN and Cr. Peak Ang‐2:Ang‐1 ratio in the first month were higher in patients with elevated ALT, AST and BUN, while sE‐selectin were higher in patients with elevated BUN and Cr. We further analysed the correlation of hepatic or renal dysfunction with endothelial cell activation in patients with CRS.

### 3.5 Endothelial cell activation is associated with hematopoietic dysfunction

**FIGURE 4:**
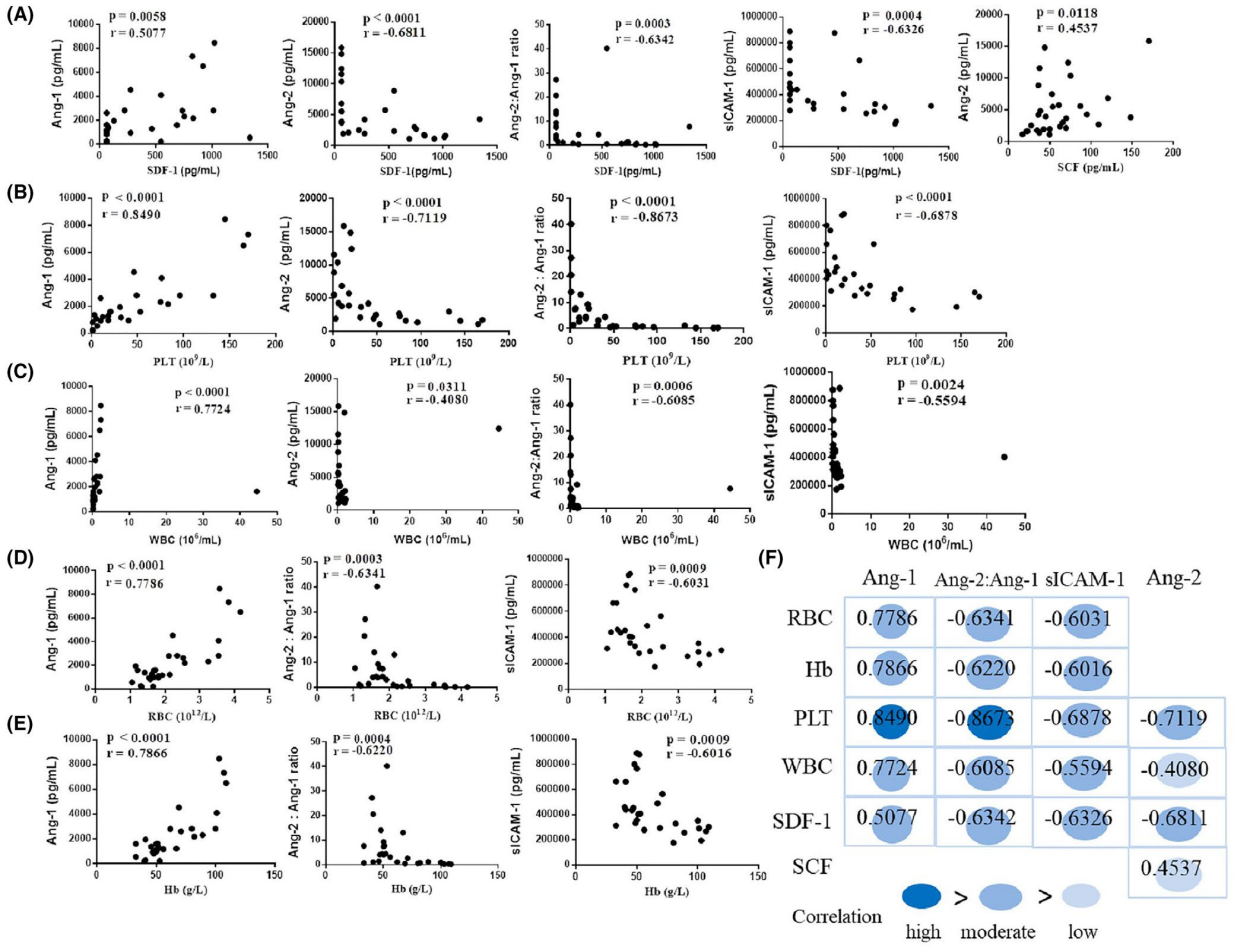
Endothelial cell activation is correlated to hematopoietic dysfunction. The correlation of serum concentration of endothelial cell activation biomarkers with the minimum SDF‐1 and SCF (A), platelet (B), white blood cell (C), red blood cell (D), haemoglobin (E) in patients with CRS (n = 28), F represents the summary of the correlations (R values) in A‐E. R values were determined using the Spearman correlation test. SDF‐1 = stromal cell–derived factor‐1, SCF = stem cell factor

Differential cell surface molecule expression between quiescent and activated endothelial cells influences not only the relative balance between pro‐ and anti‐coagulant activity, but also the degree of adhesion of circulating blood cells.^6^ We also analysed the correlation of blood counts or haematopoiesis‐related factors with the markers of endothelial cell activation in patients with CRS (Figure 4). The serum SDF‐1 level was negatively correlated with values of Ang‐2, Ang‐2:Ang‐1 ratio, sICAM‐1 and positively correlated with Ang‐1, while SCF was positively correlated with values of Ang‐2, and the correlations were moderate except SCF with Ang‐2 (Figure 4A). The PLT or WBC level was also negatively correlated with values of Ang‐2, Ang‐2:Ang‐1 ratio and sICAM, while positively correlated with Ang‐1, among which Ang‐1 or Ang‐2:Ang‐1 ratio level and PLT count was highly correlated (Spearman r > 0.8), and the others were moderately correlated except the WBC with Ang‐2 (0.3 < Spearman r ≤ 0.5) (Figure 4B‐C). The RBC or Hb level was positively correlated with ANG‐1 and negatively correlated with values of Ang‐2:Ang‐1 ratio and sICAM, and the correlations were moderate (0.5 < Spearman r ≤ 0.8) (Figure 4D‐E).

### 3.6 Endothelial cell activation‐related biomarkers could be useful as predictive biomarkers for subsequent CRS

**FIGURE 5:**
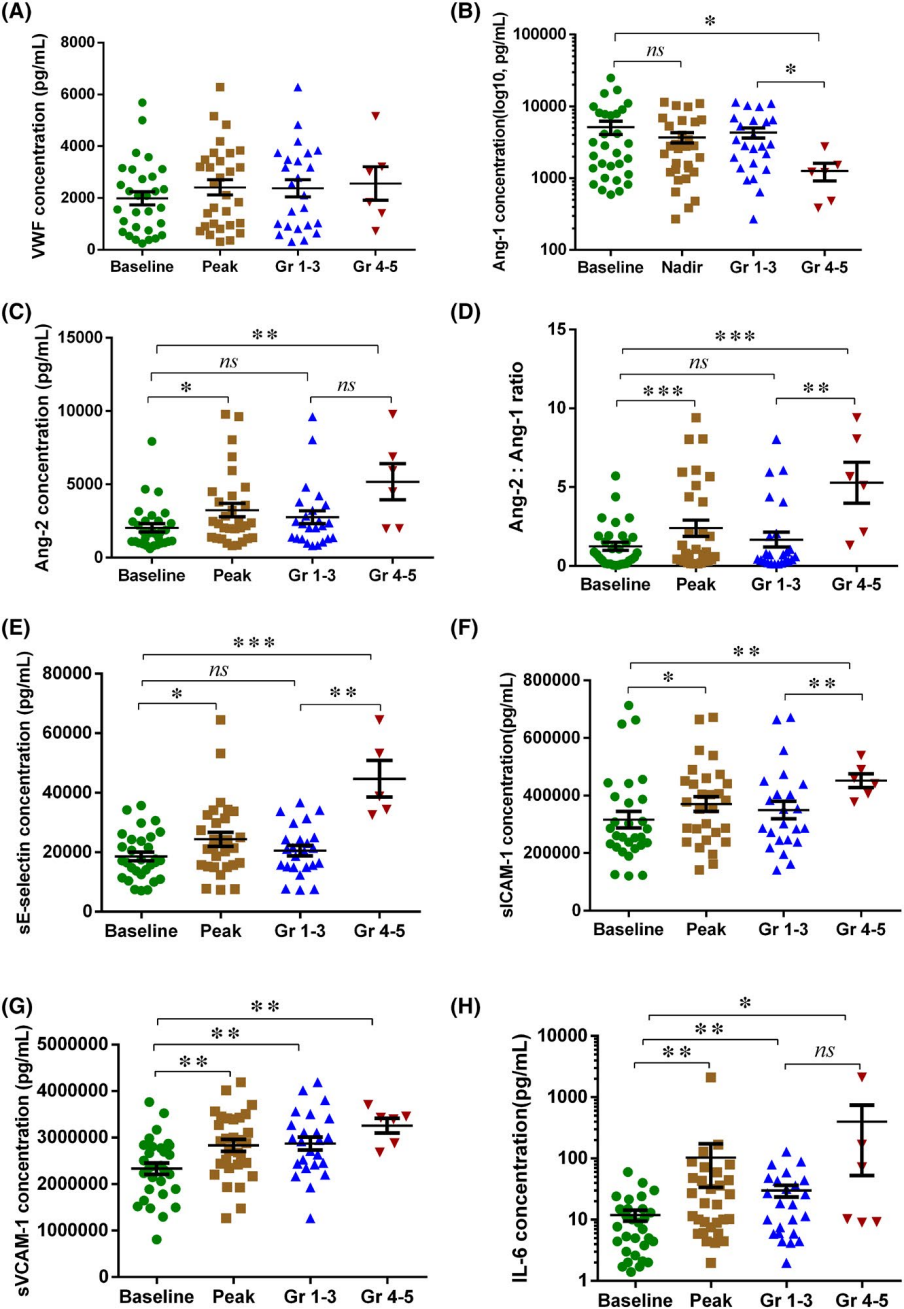
Early increases in endothelial cell‐related biomarkers after CD19 CAR‐T cells infusion are associated with severe CRS. The biomarkers were analysed from the first 3 days after infusion, before patients developed severe CRS. VWF (A), Ang‐1 (B) and Ang‐2 (C) concentrations, the Ang‐2:Ang‐1 ratio (D), sE‐selectin (E), sICAM‐1(F), sVCAM‐1(G) and IL‐6 (H) in serum collected on in the first 3 days after CAR‐T cell infusion from total patients or a subset of patients with grades 1–3 (n = 24) or grades 4–5 (n = 6) CRS. P values were determined using Student's t test for homogeneity of variance or Wilcoxon/Mann‐Whitney test for heterogeneity of variance. ^*^ *p* < 0.05,^**^ *p* < 0.01,^****^<0.001,^****^ *p* < 0.0001, ns means *p* < 0.05

**FIGURE 6:**
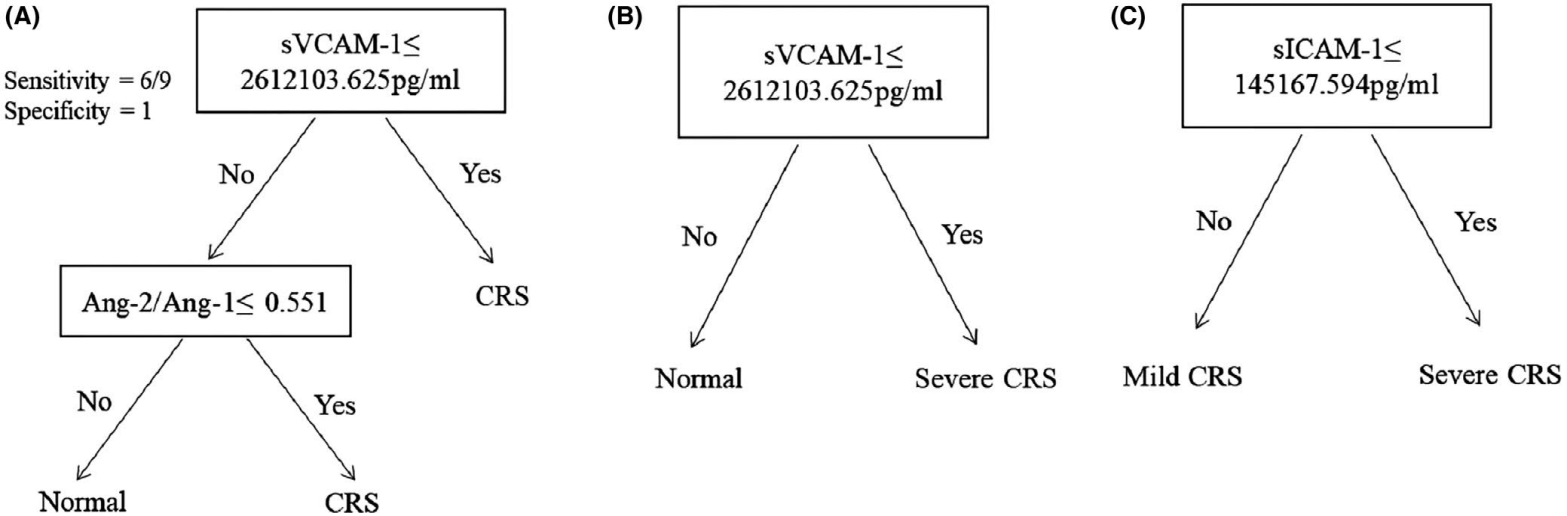
Biomarkers of endothelial activation can predict CRS. Biomarkers were analysed from the first 3 days after CAR‐T cell infusion. With a decision tree model, we could predict which patients developed CRS using sVCAM‐1 and Ang‐2:Ang‐1 ratio (A). A single biomarker sVCAM‐1 using decision tree modelling could predict the severe CRS (B). We also found sICAM‐1 could predict the severity of CRS (C)

Teachey et al pointed out that understanding the timing of the rise and fall of certain markers in patients with CRS not only helps to understand the underlying biology but also has potential therapeutic implications.^11^ Accordingly, we analysed the peak time of endothelial cell activation‐related biomarkers and CRS. The medians of VWF, Ang‐1, Ang‐2, Ang‐2:Ang‐1, sE‐selectin and sICAM‐1 peaked between days 4 and 6, which were earlier than CRS (peaked on day 8) after CAR‐T cell infusion (Table 4). Ang‐2, Ang‐2:Ang‐1, sE‐selectin, sVCAM‐1 and sICAM‐1 rised very early, and these markers were elevated before the onset of severe CRS, suggesting that the activation of endothelial cells was earlier than occurrence of CRS. Among them, Ang‐2:Ang‐1, sE‐selectin and sICAM‐1 were differently elevated for severe and mild CRS in the first 3 days after CAR‐T cell infusion (Figure 5, Supplemental Table S3). In contrast, while IL‐6 is the cytokine most strongly associated with severe CRS over the first month, early IL‐6 levels (in the first 3 days) were not different by CRS severity. With the decision tree model (Figure 6), we could predicted which patients developed CRS using sVCAM‐1 and Ang‐2:Ang‐1 with sensitivity 67% and specificity 100%. For the severe CRS, the modelling was even more accurate and we could predict which patients developed severe CRS using sVCAM‐1 only. Using sICAM‐1 only, we could also predict the severity of CRS. Together, these data indicated that biomarkers of endothelial cell activation rised early and might be useful as predictive biomarkers for subsequent CRS.

## 4 DISCUSSION

CRS is the most common and potentially life‐threatening toxicity after CAR‐T cell.^12^ Studies have identified some risk factors of developing CRS, such as higher marrow tumour burden, higher CAR‐T cell dose or severe thrombocytopenia.^13^, ^14^ The standard clinical laboratory test cytokines, such as IL‐6, CRP and ferritin in serum, are consistently found to be elevated of patients with CRS. However, a number of cytokines are not helpful in predicting CRS as they peaked after the occurrence of CRS, such as ferritin, CRP, AST, ALT, BUN and Cr.^15^ Therefore, identification of effective predictors is crucial for the early intervention and therapeutic efficiency of CRS after CAR‐T cell therapy, especially for severe CRS.

The term ‘endothelial cell activation’, represents not only a loss of vascular integrity and an anti‐thrombotic‐to‐prothrombotic phenotypic change, but also expression of adhesion molecules and cytokine production.^16^ Angiogenin (Ang)‐2 and von Willebrand factor (VWF) are stored in Weibel‐Palade bodies of endothelial cells and are released upon endothelial cell activation to promote vascular leakage, while angiogenin (Ang)‐1 mainly maintains the stability of endothelial cells.^17^ First, data from our group suggested a correlation between endothelial cell activation and CRS, manifested by elevated VWF and Ang‐2 and declined Ang‐1 concentrations in patients with CRS after CAR‐T cell therapy, which is consistent with a recent report by Hay et al.^13^ Second, we found marked differences in soluble adhesive molecules not previously studied after CAR‐T cell therapy, such as intercellular adhesion molecule‐1 (ICAM‐1) and vascular cell adhesion molecule‐1 (VCAM‐1). Expression of these adhesion molecules on the endothelial cell surface is a hallmark of endothelial cell activation.^16^ Leukocyte adhesion to endothelial cells via VCAM‐1, ICAM‐1 and E‐selectin and subsequent transendothelial migration to a site of injury or inflammation, are central features of the innate immune function of endothelial cells.^17^ These adhesion molecules, shed from activated endothelial cells and transformed into a soluble form, have been widely studied, often together, as diagnostic and prognostic markers in a variety of infectious diseases.^18^, ^19^ Third, we also compared the differences in endothelial cell activation biomarkers between patients with severe CRS and those with mild CRS, and that VWF, Ang‐1, Ang‐2, Ang‐2:Ang‐1 ratio, sE‐selectin and sICAM‐1 show a marked differences. At last, we identified endothelial cell activation‐related biomarkers that are differentially expressed in the occurrence and development of CRS, which had not been shown before. To be specific, the serum VWF, Ang‐2,sICAM‐1 and sVCAM‐1 concentrations were higher at the peak of CRS than at the start of CRS, while VWF, Ang‐1, Ang‐2, Ang‐2:Ang‐1 ratio, sE‐selectin and sICAM‐1 concentrations were reduced when CRS stopped, suggesting that the degree of endothelial cell activation was correlated with the development of CRS, which would facilitate the monitoring of patients with CRS.

After CAR‐T cell therapy, patients with CRS could exhibit liver and kidney dysfunction, as well as delayed hematopoietic recovery, characterized by elevated ALT, AST, BUN and Cr, and delayed peripheral blood recovery,^13^ but the mechanism remains unclear. Accordingly, we compared the levels of ALT, AST, BUN and Cr with endothelial cell activation markers in patients with CRS, and results showed that the serum Ang‐1, Ang‐2, Ang‐2:Ang‐1 and sICAM‐1 were increased in patients with liver function injury, and the serum VWF, Ang‐1, Ang‐2, sE‐selectin and sICAM‐1 were increased in patients with renal function injury, suggesting that biomarkers of endothelial cell activation might be involved in liver and renal function injury in CRS patients. The mechanism might be that endothelial activation/dysfunction cascade induced by the combination of elevated inflammatory cytokine levels, endothelial activation and vascular leakage leads to organ injury. Hayet al^13^ gathered marrow pathologic examination seven patients with grade ≥4 CRS and suggested that the delayed hematopoietic recovery might be attributed to the bone marrow hypocellular. Here, we evaluated the correlation of blood counts and endothelial cell activation biomarkers. Correlation analysis showed that the RBC or Hb nadirs were moderately correlated with Ang‐1, Ang‐2:Ang‐1 ratio or sICAM‐1. The PLT nadirs were highly correlated with values of Ang‐1 or Ang‐2:Ang‐1 ratio and moderately correlated with Ang‐2 or sICAM‐1. The WBC nadirs were moderately correlated with Ang‐1, Ang‐2:Ang‐1 ratio or sICAM‐1 and mildly correlated with Ang‐2. SDF‐1 is the strongest chemokine known to be involved in the homing of hematopoietic stem and progenitor cells (HSPCs).^20^ Correlation analysis showed that the serum SDF‐1 level was negatively correlated with values of Ang‐2, Ang‐2:Ang‐1 ratio, sICAM‐1 and Ang‐1, indicating that SDF‐1 level was negatively correlated with endothelial cell activation and probably thus inhibited the HSPCs homing, which is consistent with the study that the bone marrow was hypocellular in patients with grade ≥4 CRS after CAR‐T cell therapy.^13^ These data suggested that hematopoietic toxicity in CRS patients might be due to the changes of hematopoietic regulators expressed by endothelial cells, such as SDF‐1. Therefore, monitoring of endothelial cell activation biomarkers may be helpful for early interventions of liver, kidney and hematopoietic toxicity in CRS patients after CAR‐T cell therapy.

Methods to reduce the risk of CAR‐T cell therapy include identifying patients who are at high risk of developing severe CRS before therapy, modifying the treatment regimen, or early preventative interventions after CAR‐T cell infusion. We investigated if certain biomarkers of endothelial cell activation measured early could predict the occurrence and severity of CRS. As a result, we found that Ang‐2, Ang‐1, Ang‐2:Ang‐1, sE‐selectin, sVCAM‐1 and sICAM‐1 were associated with severe CRS, and Ang‐2:Ang‐1, sE‐selectin and sICAM‐1 were differently elevated in patients between severe CRS and mild CRS. Particularly, we developed decision tree models that predicted which patients were likely to develop CRS or severe CRS using endothelial activation biomarkers. With the decision tree model, we predicted which patients developed CRS using sVCAM‐1 and Ang‐2:Ang‐1 with sensitivity 67% and specificity 100%. For the severe CRS, the model was even more accurate and we could predicted which patients developed severe CRS using sVCAM‐1 only. Using sICAM‐1 only, we could identify the severity of CRS. These data indicated that the activation of endothelial cells might play a key role in CRS, and using endothelial cell activation markers as biomarkers of CRS severity or prognosis would have more clinical application value.

At present, the main view of studies hold that monocytes and macrophages are the main drivers of CRS, and the mechanism of them in CRS is as follows: after recognizing DAMP (damage‐associated molecular pattern) or PAMP (pathogen‐associated molecular pattern) moleculars by pattern recognition receptors (PRRs), they could produce inflammatory cytokines, such as IL‐1 and IL‐6, and thus participate in the occurrence and development of CRS.^21^ This study demonstrates the importance of endothelial cell activation in CRS. Endothelial cells are the first line of defence against inflammatory stress and the core regulator of cytokine storm.^22^ After activation of endothelial cells, their permeability is increased and they have a pro‐inflammatory effect.^23^ Many common and severe infectious diseases and syndromes are characterized by inflammation resulting from endothelial cell activation, and the degree of endothelial cell activation and subsequent dysfunction contribute to the severity and progression of the disease.^24^, ^25^ Here, the correlation between endothelial cell activation biomarkers and the occurrence and severity of CRS, as well as liver, kidney and hematopoietic dysfunction, confirmed the important role of endothelial cell activation in CRS. The mechanisms that lead to activation of endothelial cells maybe also due to their activating cytokines, such as IL‐6, IL‐1β, as in monocytes and macrophages. Recently, Liu et al reported that CAR‐T cell can induce the target cell pyroptosis and release DAMPs, which stimulate macrophages to release CRS‐related cytokines through the activation of the inflammasome pathway.^26^ Endothelial cells are an important part of the innate immune response since they recognize PAMPs through expression of PRRs such as TLR (Toll‐like receptor) and NLR (Nod‐like receptor), and they might also might participate in CRS through the activation of the inflammasome pathway after CAR‐T cell infusion.^27^

## CONFLICT OF INTEREST

The authors have declared that no conflict of interest exists.

## AUTHOR CONTRIBUTIONS

**Fei Hong:** Conceptualization (supporting); Data curation (supporting); Formal analysis (lead); Investigation (supporting); Methodology (equal); Project administration (equal); Resources (supporting); Software (equal); Supervision (supporting); Validation (equal); Visualization (equal); Writing‐original draft (lead). **Ming Shi:** Conceptualization (equal); Data curation (equal); Investigation (equal); Project administration (equal); Resources (lead); Supervision (equal); Writing‐original draft (supporting). **Jiang Cao:** Conceptualization (supporting); Data curation (supporting); Investigation (supporting); Project administration (supporting); Resources (equal); Supervision (supporting); Writing‐original draft (supporting). **Ying Wang:** Data curation (supporting); Formal analysis (supporting); Methodology (supporting); Project administration (supporting); Resources (supporting); Supervision (supporting); Validation (supporting); Visualization (supporting). **yanqing gong:** Data curation (equal); Formal analysis (supporting); Resources (supporting). **Hui Gao:** Formal analysis (supporting); Resources (supporting); Validation (supporting). **li zhenyu:** Investigation (supporting); Supervision (supporting). **Junnian Zheng:** Conceptualization (supporting); Validation (supporting). **Ling yu Zeng:** Conceptualization (equal); Funding acquisition (supporting); Project administration (supporting); Supervision (supporting). **Aili He:** Conceptualization (supporting); Data curation (supporting); Funding acquisition (equal); Project administration (supporting); Supervision (equal); Writing‐review & editing (supporting). **Kailin Xu:** Conceptualization (lead); Funding acquisition (lead); Investigation (equal); Project administration (lead); Resources (lead); Supervision (equal).

## ETHICAL APPROVAL

This study was performed in accordance with the Declaration of Helsinki and was approved by the Ethics Committee of the Affiliated Hospital of Xuzhou Medical University. All patients provided written informed consent.

## Supporting information

Table S1‐S3

## Data Availability

The data will be available when all primary and secondary endpoints have been met, except data that involve privacy or protection. Any application for data will be reviewed by the experiment manager. Access to data will be given for applicants that reasonably use the data and experimental methods. To gain access, requestors will also need to sign a data access agreement.
