# Supplementary material for: Predictive role of endothelial cell activation in cytokine release syndrome after chimeric antigen receptor T cell therapy for acute lymphoblastic leukaemia
Source: J Cell Mol Med. 2021 Nov 3;25(24):11063–74. doi: 10.1111/jcmm.17029 (PMC8650023; doi:10.1111/jcmm.17029)
Supplement: Supplementary file 1 — Table S1‐S3 [file JCMM-25-11063-s001.docx]

**Supplemental Table 1. Median (range) for baseline values of endothelial cell-related markers** **(pg/mL) are presented for study subjects (N = 30) and normal controls (N = 7). Baseline values (pg/mL) associated with endothelial cell activation compared to normal donors.**

|  | **Normal controls (N =7)** | **Study Subjects (N =30)** |
| --- | --- | --- |
| **VWF** | 872.1(218.2 - 1447) | 1852 (244.3 - 5683)* |
| **ANG1** | 8044(3385 - 19624) | 2838(591.3 - 24880)* |
| **ANG2** | 1022(477.4- 2527) | 1066(606.3- 7929) |
| **ANG2:ANG1** | 0.099 (0.045- 0.454) | 0.777 (0.0353- 5.707)* |
| **sE-selectin** | 18847(12160 - 24343) | 17200(7106 - 35671) |
| **sVCAM-1** | 1.096e+006(597349 -2343954) | 2.383e+006 (810362-3763817)** |
| **sICAM-1** | 206692(121288- 633727) | 284506(121084- 808739)* |
| **SCF** | 24.27(18.67- 156.8) | 60.53(20.32- 234.7)* |

Baseline cytokine values for study subjects (N=30) were compared to 7 normal controls using the exact Wilcoxon test. Significance testing was done at the 0.05 level. Baseline was defined as the value observed within 7 days prior to and closest to day of infusion. * p < 0.05, ** p < 0.01

**Supplemental Table 2. Median (Range) for one-month-peak/nadir biomarker values (pg/mL) in ALL patients after CD19-targeted CAR-T therapy (N = 30)**

|  | **Grade 1-3 ( N =24 )** | **Grade 4-5 ( N =6 )** |
| --- | --- | --- |
| **VWF** | 2445 (633.6 - 4827) | 4916 (3506 - 5386)** |
| **Ang-1 nadir** | 2068 (558.2 - 8469) | 1019 (200.8 - 1599)* |
| **Ang-2** | 2823 (1102 - 12447) | 10221 (5469 - 15857)*** |
| **Ang-2:Ang-1** | 1.116 (0.173 - 8.365) | 13.56 (4.434 - 40.29)**** |
| **sE-selectin** | 20690 (7335 - 41743) | 34387 (27460 - 89451)** |
| **sICAM-1** | 302376 (141744 - 671825) | 567387 (354389 -887997)** |
| **sVCAM-1** | 4.457e+006 (2568570-6369013) | 3.454e+006 (2683702-5361721) |

Peak/nadir biomarker values within one month were compared between those who developed severe (grade 4-5) CRS versus mild (grade 1-3) CRS using Mann-Whitney test. * p < 0.05, ** p < 0.01, *** p < 0.001, **** p < 0.0001

**Supplemental Table 3. Median (Range) for day 0-3 day peak/nadir biomarker values (pg/mL) in ALL patients after CD19-targeted CAR-T therapy (N = 30)**

|  | **Grade 1-3 ( N =24 )** | **Grade 4-5 ( N =6 )** |
| --- | --- | --- |
| **IL-6** | 19.58 (1.67 - 129.0) | 73.85 (9.16 - 2121) |
| **VWF** | 1804 (307.9 - 4827) | 3965 (1410 - 5159) |
| **ANG1** | 2822 (270.5 - 11478) | 1019 (385.7 - 2756)* |
| **ANG2** | 2823 (1102 - 12447) | 10221 (5469 - 15857) |
| **ANG2:ANG1** | 0.714 (0.129 - 8.034) | 6.582 (1.308 - 9.411)** |
| **sE-selectin** | 26733 (10745 - 51088) | 45709 (32542 - 89451)** |
| **sVCAM-1** | 2.676e+006 (1265863-4190675) | 3.440e+006 (2683702-5361721) |
| **sICAM-1** | 352952(175271 - 688023) | 723214(354389 -887997)** |

Peak/nadir biomarker values within 3 days were compared between those who developed severe (grade 4-5) CRS versus mild (grade 1-3) CRS using Mann-Whitney test. * p < 0.05, ** p < 0.01
